# Infusion of Megakaryocytic Progenitor Products Generated from Cord Blood Hematopoietic Stem/Progenitor Cells: Results of the Phase 1 Study

**DOI:** 10.1371/journal.pone.0054941

**Published:** 2013-02-04

**Authors:** Jiafei Xi, Honghu Zhu, Daqing Liu, Xue Nan, Wen Zheng, Kaiyan Liu, Wei Shi, Lin Chen, Yang Lv, Fang Yan, Yanhua Li, Xiaoyan Xie, Yunfang Wang, Wen Yue, Xin Xu, Xiaofei Wei, Jun Zhu, Xiaojun Huang, Xuetao Pei

**Affiliations:** 1 Stem Cell and Regenerative Medicine Lab, Beijing Institute of Transfusion Medicine, Beijing, China; 2 Peking University People’s Hospital, Peking University Institute of Hematology, Beijing, China; 3 Key Laboratory of Carcinogenesis and Translational Research (Ministry of Education), Peking University Cancer Hospital and Institute, Beijing, China; 4 Beijing Cord Blood Bank, Beijing, China; B.C. Cancer Agency, Canada

## Abstract

**Background:**

Currently, a constant shortage in the supply of platelets has become an important medical and society challenge, especially in developing country, and the *in vitro* production of megakaryocytic progenitor cells (MPs) from cord blood could represent an effective platelet substitute. In the present study, our objective was to determine the safety and feasibility of *ex vivo* generated MPs in patients.

**Methods and Findings:**

MPs were produced and characterized from cord blood mononuclear cells under a serum free medium with cytokines. We investigated the feasibility of expansion and infusion of cord blood-derived MPs in 24 patients with advanced hematological malignancyes. The primary end point was the safety and tolerability of the infusion of cord blood-derived MPs. No adverse effects were observed in patients who received *ex vivo*-generated cells at concentrations of up to a median value of 5.45×10^6^cells/kg of body weight. With one year follow-up, acute and chronic GVHD had not been observed among patients who received MPs infusion, even without ABO blood group and HLA typing matching.

**Conclusions:**

These initial results in patients are very encouraging. They suggest that infusion of cord blood-derived MPs appears safe and feasible for treatment of thrombocytopenia.

**Trial Registration:**

www.chictr.org ChiCTR-TCH-09000333.

## Introduction

Thrombocytopenia is a common and potentially fatal complication of chemotherapy and hematopoietic stem cell transplantation. Infusion of platelets from unrelated donors is currently the only effective treatment to prevent fatal hemorrhage. However, owing to their short storage time and increased demand, a constant shortage in the supply of platelets has become an important medical and society challenge. [1] Therefore, investigation of alternative sources of platelets would be beneficial.

Hematopoietic stem cells (HSCs) can be used to generate functional megakaryocytic progenitors (MPs), megakaryocytes, and platelets on a large scale. [2], [3], [4] Functional MPs and platelets have successfully been produced *in vitro* from CD34^+^ hematopoietic cells from bone marrow, cord blood, and peripheral blood. [5], [6], [7] Several studies have reported that transplantation of *in vitro* auto-producing MPs can promote platelet recovery after high-dose therapy and HSC transplantation. [8], [9].

Umbilical cord blood is an abundant source of HSCs. [10], [11] Furthermore, cord blood is highly enriched in committed hematopoietic progenitor cells, including those of the megakaryocytic lineage. *In vitro* large scale production of MPs from cord blood could represent an effective platelet substitute. *In vitro* cord blood-derived MPs can be used in many non-emergency conditions, allowing platelets to be used for the emergency conditions. However, there are no reports in the literature that document the safety and efficacy of *ex vivo*–expanded cord blood-derived MPs in clinical trials.

Following high-dose chemotherapy, neutropenia and thrombocytopenia places patients at an increased risk of developing severe infectious and bleeding complications. The transplantation of *ex vivo*-generated hematopoietic progenitor cells in addition to chemotherapy represents a possible treatment for high-dose chemotherapy-induced cytopenia. Theoretically, the additional transplantation of *ex vivo* generated progenitor and post-progenitor cells might lead to the production of sufficient numbers of mature functional cells within a few days after transplantation. The feasibility and efficacy of this approach with regard to neutrophil recovery has previously been demonstrated. [12], [13] However, potential clinical benefits of transplanted cord blood derived MPs has not yet been shown.

We successfully generated MPs from cord blood using a combination of cytokines. Based on promising results of our preclinical study, state food and drug administration (SFDA) of China approved our group to conducts a clinical trial of MP injection to patients with hematologic malignancy. We report the results of our phase 1 study of cord blood-derived MPs in 24 patients with hematological malignancy. We believe this to be the first report of MPs from cord blood in a human clinical trial.

## Methods

### Trial Protocols

The protocols for this trial are available as supporting information; see Protocol S1 and S2.

### Ethics Statement

The study was approved by Research Ethics Committee of Peking University Cancer Hospital & Institute and by the SFDA of China. All patients provided written informed consent in accordance with the principles of the Declaration of Helsinki before enrollment. We obtained informed written consent from the next of kin, parents or guardians on the behalf of the minors/children participants involved in our study.

### Patient Inclusion and Exclusion Criteria

Twenty-four consenting patients with advanced hematological malignancy were enrolled in this study. The target size of our study is 20–30 patients. Patients with hematological malignancy were eligible to receive MPs if they met the following criteria: 12–60 years of age; post-chemotherapy; 20≤PLT≤60 (×10^9^/L); no serious damage to liver and kidney function; written informed consent. The exclusion criteria: thrombocytopenia without cancer chemotherapy; patients with primary disease in important organs (liver, kidney), patients accompanied by the immune system disease, serious infections, mental disorders or organ transplant recipients; pregnant, breast-feeding women; patients did not sign informed consent form; infusion contraindication about injection of umbilical cord blood MPs.

### Study Design

**Figure 1:**
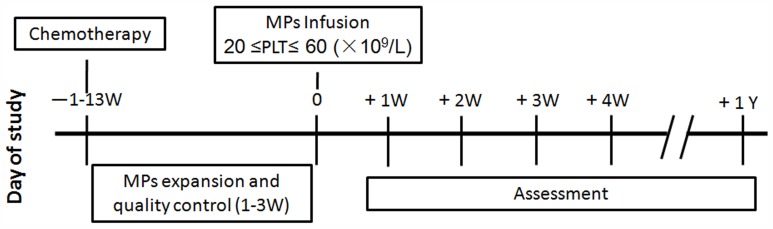
Study flow chart.

This was a single-center, open-label, phase 1 clinical trial designed to assess the safety profile of infusion of *ex vivo*-expanded cord blood-derived MPs after chemotherapy. MPs from cord blood were infused when the platelet of patients was 20≤PLT≤60 (10^9^/L) after chemotherapy, and the toxicity was evaluated. The interval time between the cord blood MPs preparation and patient infusion was two hours. Before MP infusion, the patients was given 10 ml 10% calcium gluconate to prevent anaphylaxis. The overall infusion time was within two hours. Platelet counts were assessed at least twice per week. After treatment with cord blood-derived MPs, we conducted one-year follow-up. The study flow chart is depicted in Figure 1. This study was registered with http://www.chictr.org, number ChiCTR-TCH-09000333.

### In vitro Suspension Culture

After informed consent had been obtained, cord blood from healthy volunteers was collected into sterile heparinized tubes. Mononuclear cells were obtained by centrifugation on Lymphoprep density gradient. Ex vivo cultures were set up with cord blood MNCs at 3×10^6^ cells/mL in StemSpan medium supplemented with 50 ng/mL thrombopoietin (TPO) (Shenyang Sunshine Pharmaceutical Company Limited), 20 ng/mL interleukin-3 (IL-3) (Peprotech), 50 ng/mL stem cell factor (SCF) (Peprotech) and 50 ng/ml IL-6 (Peprotech) using 75 cm^2^ tissue culture flasks (Corning). At indicated time points, the cultured cells were harvested, washed, and prepared for immediate infusion.

### Flow Cytometry Analysis

*Ex vivo*-generated cells were characterized by flow cytometry on day 0, 7, 10, and 14. All of the conjugated antibodies and the corresponding isotype controls were purchased from BD Pharmingen. The antibodies used were CD3, CD14, CD19, CD41, CD56, and CD61. MPs were collected and washed twice in PBS with 0.1% BSA and stained in accordance with the manufacturer’s suggested concentration of conjugated antibody for 30 min at 4°C. The stained cells were then washed twice in PBS with 0.1% BSA and fixed with the wash buffer supplemented with 1% paraformaldehyde. The samples were then analyzed using a flow cytometer (Becton Dickinson). Cell populations were analyzed with the CellQuest program(Becton Dickinson). Three experiment were performed. We also analyzed the cell size and the co-staining of CD41 on the cultured MPs.

### Colony Forming Unit-MK Assay

To demonstrate Colony forming unit-MK colonies, 5×10^4^ *in vitro* cultured cells were cultured with MegaCult-C media (StemCell Technologies) in the presence of 10 ng/mL recombinant human IL-3 (Peprotech), 20 ng/mL recombinant human IL-6 (Peprotech), and 50 ng/mL recombinant human TPO (Shenyang Sunshine Pharmaceutical Company Limited) according to the instruction manual. After 10–14 days, the cells were fixed, dried, and stained with an anti-CD41 specific antibody or isotype control antibody according to the manufacturer’s instructions. CFU-MK colonies were defined as colonies with at least 3 megakaryocytes. Three experiment were performed.

### Megakaryocytes Analysis

For morphological analysis of mature megakaryocytes in the culture, day 14 cultured cells were centrifuged using a cytospin centrifuge. Slides were then stained with Wright-Giemsa. We also analyzed the platelets generation in the day 14 culture by flow cytometry. Culture-derived platelets were enumerated as CD41^+^ events with the same scatter properties as blood platelets. Ten thousand events were counted per sample. Three experiment were performed.

### End Points and Definitions

The primary end point was the safety and tolerability of the infusion of cord blood-derived MPs. Patients were monitored after the infusion of MPs for infusional toxicity and adverse events, which were evaluated according to World Health Organization (WHO) criteria. Secondary end points included platelet recovery after infusion of MPs. The ABO blood group and HLA typing of patients and paired cord blood were carried out at Department of hematology, Peking University People’s Hospital, (Beijing, China).

## Results

### Patients and MP Characteristics

Twenty-four patients (nine females/fifteen males) were treated between March 2009 and May 2010. The study flow chart is depicted in Figure 1. The median age was 37 years (range, 15–65 years), and the median weight was 68.5 kg (range, 50–92 kg). The patient group included 22 patients with non-Hodgkin’s lymphoma (NHL), one patient with Hodgkin’s lymphoma (HL), and one patient with multiple myeloma (MM). The median number of previous chemotherapy courses were of five (range, 1–17), and the median interval time between the last chemotherapy and MP infusion was 14 days (range, 3–91 days). Baseline characteristics are presented in Table 1.

**Table 1:** Patient characteristics.

No	Age	Gender	Diagnosis	Time ofDiagnosis	Chemotherapycourses	LastChemotherapy	Recruitmenttime	Intervaltime	Chemotherapyregimen
1	39	F	NHL IVA	2008-6-26	9	2009-4-7	2009-4-14	8	GEMOX+ENDOSTAR
2	21	M	NHL IVB	2008-9-12	10	2009-5-5	2009-5-19	14	DICE+ ENDOSTAR
3	29	M	HL IVB	1992-6-14	10	2009-6-1	2009-6-26	26	AraC
4	56	M	NHL IVB	2009-2-16	7	2009-6-22	2009-7-10	19	DICE+ ENDOSTAR
5	26	M	NHL IVB	2009-5-4	3	2009-7-8	2009-7-21	14	CAT
6	62	F	MM III	2009-7-9	1	2009-7-19	2009-7-30	12	BTD
7	47	F	NHL IVB	2009-6-23	1	2009-7-30	2009-8-7	9	R-CHOP
8	40	M	NHL IVB	2009-8-11	3	2009-8-11	2009-8-21	11	MTX
9	30	M	NHL IVA	2009-4-17	5	2009-8-28	2009-9-22	26	VALP
10	65	F	NHL IVB	2004-9-16	16	2009-9-9	2009-10-16	37	FC
11	19	F	NHL IVB	2009-7-16	5	2009-10-14	2009-10-28	15	DICE+ ENDOSTAR
12	35	M	NHL	2009-11-4	1	2009-11-4	2009-11-12	9	VDLP
13	27	M	NHL IVB	2009-10-29	1	2009-11-6	2009-11-20	15	VDLP
14	45	M	NHL IVA	2008-2-26	17	2009-11-25	2009-12-7	14	AraC+ATO+Tioprinin
15	45	M	NHL IVA	2008-5-21	9	2009-12-13	2009-12-15	3	GEMOX
16	31	F	NHL IVA	2007-12-26	16	2009-12-14	2009-12-26	13	VDLP
17	57	F	NHL IVB	2009-7-1	5	2009-12-12	2010-1-15	33	RFC
18	59	F	NHL IIIB	1992-4-10	17	2010-1-14	2010-1-26	13	DICE+ ENDOSTAR
19	48	M	NHL IIIB	2009-11-18	3	2010-1-25	2010-2-9	16	EP
20	31	M	NHL IA	2009-12-14	4	2010-3-12	2010-3-23	12	MTX
21	50	F	NHL IVA	2009-8-7	6	2010-1-15	2010-4-16	91	FC
22	15	M	NHL IVA	2010-1-25	1	2010-4-10	2010-4-21	11	BFM-90
23	32	M	NHL IVA	2009-10-12	6	2010-4-19	2010-5-9	20	MTX
24	45	M	NHL IIIB	2007-2-1	9	2010-3-31	2010-5-24	54	R-COP

Our preclinical studies had established the method to efficiently inducing and expansing cord blood mononuclear cells (MNCs) to MPs. The in vitro producing MPs were characterized by morphological observation, flow cytometry analysis, and colony formation detection. Cell morphology changed obviously with the extension of culture time (Figure S1A). Flow cytometry analysis showed that the expression of CD41 (a MPs marker, Figure S1E) and CD61 (MPs, Figure S1G) was greater at day 14 than day 0 (7.9 fold and 11.6 fold), while the expression of CD3 (T cells, Figure S1B), CD19 (B cells, Figure S1D), CD56 (NK cells, Figure S1F) was lower at day 14 than day 0 (6.7 fold, 95 fold and 17.5 fold). And the expression of CD14 (monocytes, Figure S1C) was also lower at day 14 than day 0 (1.6 fold). Expansion of CFU-Mk were compared in different culture time. Ex vivo expansion achieved a 64±7 fold increase in CFU-Mk by 14 days culture (Figure S1H). Morphological features of day 14 cultured cells and the presence of polyploid nuclei were confirmed by microscopy (Figure S1I). This results showed that the *ex vivo*-generated-MPs can mature to megakaryocytes at day 14 culture. Although, the number of mature megakaryocytes were still low. And we also showed that the MKs in culture produced platelets. The platelets in culture were enumerated by flow cytometry as particles having the same scatter properties as blood platelets and expressing CD41 antigen. The number of platelets released remained relatively low with a mean of 5.5±0.9% of the total expanded population on day 14. Moreover, we analyzed the cell size and co-staining of CD41. And the results of a representative experiment was shown in Figure S2.

Twenty-four umbilical cord blood units identified for MP manufacture contained a median of 8.55×10^7^ MNCs (range, 1.9–29 cells) and a median of 76 mL (range 47–106 mL). The results of *ex vivo* expansion culture are summarized in Table 2. The median expansion was 73.5-fold (range, 3-365-fold). After expansion culture, the median proportion of CD41^+^_cells was 35% (range, 8%–77%). The dose of infused MPs for individual patients is summarized in Table 2.

**Table 2:** *Ex vivo* expansion: Summary of product characteristics before and after expansion.

NO.	Before expansion				After expansion					Composition of Transplanted Grafts		
	Volume of CB (ml)	MNC×10^7^	% CD41^+^	Expansion time (day)	MNC×10^7^	% CD41^+^	AbsoluteCD41^+^(×10^6^)	Cell Viability	Fold expansion	Body Weight	MNC per kg b.w. (×10^6^)	CD41^+^ cells per kg b.w. (×10^6^)
1	78	4.5	6	11	58.5	52	304	91.4%	113	70	8.4	4.3
2	54	8	11	5	48	26	125	94.0%	14	61	7.9	2.0
3	69	24	4	7	264	33	871	98.7%	91	67	39.4	13.0
4	47	4.6	8	6	611	22	1344	98.0%	365	57	107.2	23.6
5	93	8.1	3	8	72.9	27	197	98.9%	81	92	7.9	2.1
6	82	16	8	7	128	22	282	99.0%	22	54	23.7	5.2
7	106	18	6	4	54	13	70	99.9%	6	60	9.0	1.2
8	52	4.6	9	17	87.4	64	559	97.1%	135	81	10.8	6.9
9	77	1.9	13	20	32.3	55	178	92.2%	7	67	4.8	2.7
10	62	6	6	4	36	9	32	99.0%	9	57	6.3	0.6
11	70	5	12	12	130	72	936	87.8%	156	79	16.5	11.8
12	77	7	5	14	119	67	797	93.0%	228	67	17.8	11.9
13	72	10	6	4	20	8	16	95.0%	3	63	3.2	0.3
14	78	9	9	12	198	77	1525	91.0%	188	70	28.3	21.8
15	86	10	8	14	310	54	1674	84.4%	209	86	36.0	19.5
16	99	15	6	7	120	23	276	93.7%	31	82	14.6	3.4
17	56	11	5	4	44	13	57	98.0%	10	78	5.6	0.7
18	93	9	5	8	135	37	500	84.0%	111	80	16.9	6.2
19	89	12	8	13	192	58	1114	95.1%	116	66	29.1	16.9
20	70	20	5	13	380	63	2934	92.0%	293	80	47.5	29.9
21	72	6	9	22	96	32	307	94.2%	57	50	19.2	6.1
22	102	29	6	6	132	18	238	93.9%	14	88	15.0	2.7
23	65	6.6	11	16	85	52	442	92.0%	61	78	10.9	5.7
24	75	5.1	8	11	56	48	269	90.2%	66	61	9.2	4.4
Median(range)	76(47–106)	8.55(1.9–29)	7(3–13)	9.5(4–22)	107.5(20–611)	35(8–77)	305.5(16–2934)	93.95%(84–99.90%)	73.5(3–365)	68.5(50–92)	14.8(3.2–107.2)	5.45(0.3–29.9)

CB, cord blood; MNC: mononuclear cells.

### MP Infusion Toxicity Profile

The infusion toxicity profile is summarized in Table 3. Severe adverse events associated with infusion of cultured cord blood-derived MPs were not observed. Two patients experienced fever (<38°C), four patients exhibited increased transaminases, one patient experienced increased jaundice (jaundice was present prior to infusion of MPs), and one patient experienced vomiting. All conditions were resolved with standard clinical management. With regard to four patients with elevated transaminases, we recorded the transaminase changes before and after the MP infusion. The results showed that all four patients experienced increased transaminitis prior to infusion of MPs. (Figure S3) In the present clinical trial, we did not carry out the ABO blood group and HLA matching. However, after one year follow-up acute and chronic GVHD had not been observed among patients who received MPs infusion. The ABO blood group and HLA typing data of patients and paired cord blood were shown in Table S1.

**Table 3:** Adverse reaction in MP-infusion patients.

Adverse events	No	Patient Number
**Fever**	2	10, 15
**Anaphylaxis**	0	NA
**Hemolysis**	0	NA
**Shock**	0	NA
**Embolism**	0	NA
**Hepatolienomegaly**	0	NA
**Abnormal electrocardiogram**	0	NA
**Transaminitis**	4*	1, 2, 7, 20
**Abnormal renal function**	0	NA
**Jaundice**	1†	7
**Vomiting**	1	20
**Diarrhea**	0	NA
**Erythra**	0	NA
**GVHD**	0	NA

* All four patients exhibited increased transaminitis before infusion of MPs.

† Jaundice existed before infusion of MPs.

### Clinical Reactions

The median dose of infused MPs was 14.8×10^6^ MNCs/kg body weight (range, 3.2×10^6^−107.2×10^6^), and 5.45×10^6^ CD41^+^ cells/kg body weight (range 0.3×10^6^−29.9×10^6^). Platelet recovery occurred in 17 patients with a median platelet recovery >60×10^9^/L on day 3 (median, 1 to 16 days) and in 13 patients with a median platelet recovery >100×10^9^/L on day 6 (median, 3 to 14 days). However, 5 patients received transfusion of one unit of platelets after infusion of MPs. In the absence of platelet transfusion, platelet recovery was prompt in 12 patients with a median platelet recovery >60×10^9^/L on day 3 (median, 1 to 11 days), and 11 patients with a median platelet recovery >100×10^9^/L on day 6 (median, 3 to 14 days). The summary of platelet recovery after infusion of MPs is shown in Table 4. However, it was difficult to determine whether there is any benefit to the administration of MPs after chemotherapy without appropriate controls. And definitive results will require the completion of a randomized phase 2 clinical trial.

**Table 4:** Platelet recovery parameters after infusion of *ex vivo*-generated cells.

Patient No	PLT Day 0×10^9^/L	Platelets (PLT)			Number of PLT transfusions
		Days with PLT	Days to PLT		
		Less than 20×10^9^/L	More than 60×10^9^/L	More than 100×10^9^/L	
**1**	52	13	NA	NA	0
**2**	57	NA	4	8	0
**3**	46	NA	2	5	1
**4**	49	3	2	6	0
**5**	45	4	NA	NA	0
**6**	37	NA	3	5	0
**7**	4	0	10	NA	1
**8**	49	NA	10	NA	0
**9**	53	NA	2	7	0
**10**	56	NA	3	NA	0
**11**	56	NA	1	3	0
**12**	53	NA	2	5	0
**13**	51	NA	11	14	0
**14**	24	NA	7	14	0
**15**	24	2	NA	NA	1
**16**	63	4	NA	NA	1
**17**	41	NA	16	NA	0
**18**	23	NA	NA	NA	1
**19**	30	NA	2	6	0
**20**	46	NA	3	5	0
**21**	25	NA	NA	NA	0
**22**	35	NA	10	12	0
**23**	44	NA	4	6	0
**24**	47	NA	NA	NA	0

NA, not applicable.

## Discussion

To our knowledge, this is the first report of the use of *ex vivo*–expanded cord blood-derived MPs in humans. We investigated the feasibility of *in vitro* expansion and transplantation of cord blood-derived MPs in 24 patients with advanced hematological malignancy. Administration of *ex vivo*-generated cell numbers up to a median of 5.45×10^6^/kg body weight was performed, with no adverse effects. Administration of cord blood-derived MPs appears to be safe and feasible for treatment of thrombocytopenia after chemotherapy.

The additional transplantation of *ex vivo*-generated cell populations containing up to a median of 14.8×10^6^ MNCs/kg (range, 3.2×10^6^−107.2×10^6^), and a median of 5.45×10^6^ CD41^+^ cells/kg (range, 0.3×10^6^−29.9×10^6^), is comparable to the number of additionally transplanted megakaryocytic in previous studies. [8], [9] Our present study showed that there were no significant detriment to the administration of dose escalated MP. Now, many studies try to improve the efficiency of MP differentiation and expansion from stem cells. [14], [15], [16] The most recent studies also show that platelets can be efficiently produced from pluripotent stem cell *in vitro*. [17]–[20] All of these studies indicate promise for *in vitro*-produced platelet substitutes from stem cells.

Our study hadn’t showed definitive efficacy of MP infusion on thrombocytopenia after chemotherapy. Other similar clinical studies have used autologous MPs or megakaryocyte infusion; however, these studies also lacked a control group, and thus cannot demonstrate the exact effect of infusion of *ex-vivo*-expanded studies. [2], [3], [8], [9] Platelet recovery after chemotherapy can be influenced by patient condition, different chemotherapy regimens, and dosage and opportunity of infusion. Therefore, the efficacy of infusion of cord blood-derived MPs needs to be verified by larger phase 2 clinical trials.

There are limits in our present study. The efficiency of *in vitro* expansion should be improved, and platelet recovery effects need to be further explored by phase 2 study. In our study, patients had advanced hematological malignancy, and repeated chemotherapy can result in damage to the hematopoietic microenvironment, possibly influencing the effects of MP infusion.

In conclusion, this study demonstrates the safety of *ex vivo*-expanded MPs from CB infused after chemotherapy in patients with hematological malignancy. Our study also demonstrated the feasibility of manufacturing 3×10^8^ MPs from cord blood units to provide a cell dosage of 3.75×10^6^ cells/kg for an 80-kg recipient. However, definitive efficacy will require the completion of a randomized phase 2 clinical trial.

## Supporting Information

Figure S1 **Characterization of megakaryocytic progenitors from cord blood mononuclear cells.** (A) Cell morphology of different culture time by microscope observation. Scale bars: 100 µm. Surface marker expression of CD3 (B), CD14 (C), CD19 (D), CD41 (E), CD56 (F), and CD61 (G) in expanded CB population during 14 days of culture. (H) CFU-Mk expansions after 14 days of culture. Data are shown as mean±SD from three experiments. (I) Typical morphology of mature megakaryocytes on day 14 culture was shown. Scale bars: 50 µm.
(TIF)

Figure S2 **FACS plots showed the cell size and co-staining of CD41^+^ cells at day 14 culture.** (A) and (B) showed the results of isotype IgG. (C) and (D) showed the results of CD41 expression.
(TIF)

Figure S3 **Transaminase changes of four patients before and after MPs infusion.** (A) Data of patient 1. The chemotherapy time is 2009/4/7, and the MPs infusion time is 2009/4/14. (B) Data of patient 2. The chemotherapy time is 2009/5/5, and the MPs infusion time is 2009/5/19. (C) Data of patient 7. The chemotherapy time is 2009/7/30, and the MPs infusion time is 2009/8/7. (D) Data of patient 20. The chemotherapy time is 2010/3/12, and the MPs infusion time is 2010/3/23. All the four patients had different degrees of elevated transaminases before MP treatment. Dashed lines shows the normal value of ALT and AST. ALT: alanine transarninase, AST: aspartate aminotransferase.
(TIF)

Table S1 **ABO blood group and HLA typing data of patients and paired cord blood.**
(DOC)

Protocol S1 (DOC)

Protocol S2 (DOC)
