# Supplementary figures and images for: Infusion of Megakaryocytic Progenitor Products Generated from Cord Blood Hematopoietic Stem/Progenitor Cells: Results of the Phase 1 Study
Source: PLoS One. 2013 Feb 4;8(2):e54941. doi: 10.1371/journal.pone.0054941 (PMC3563646; doi:10.1371/journal.pone.0054941)

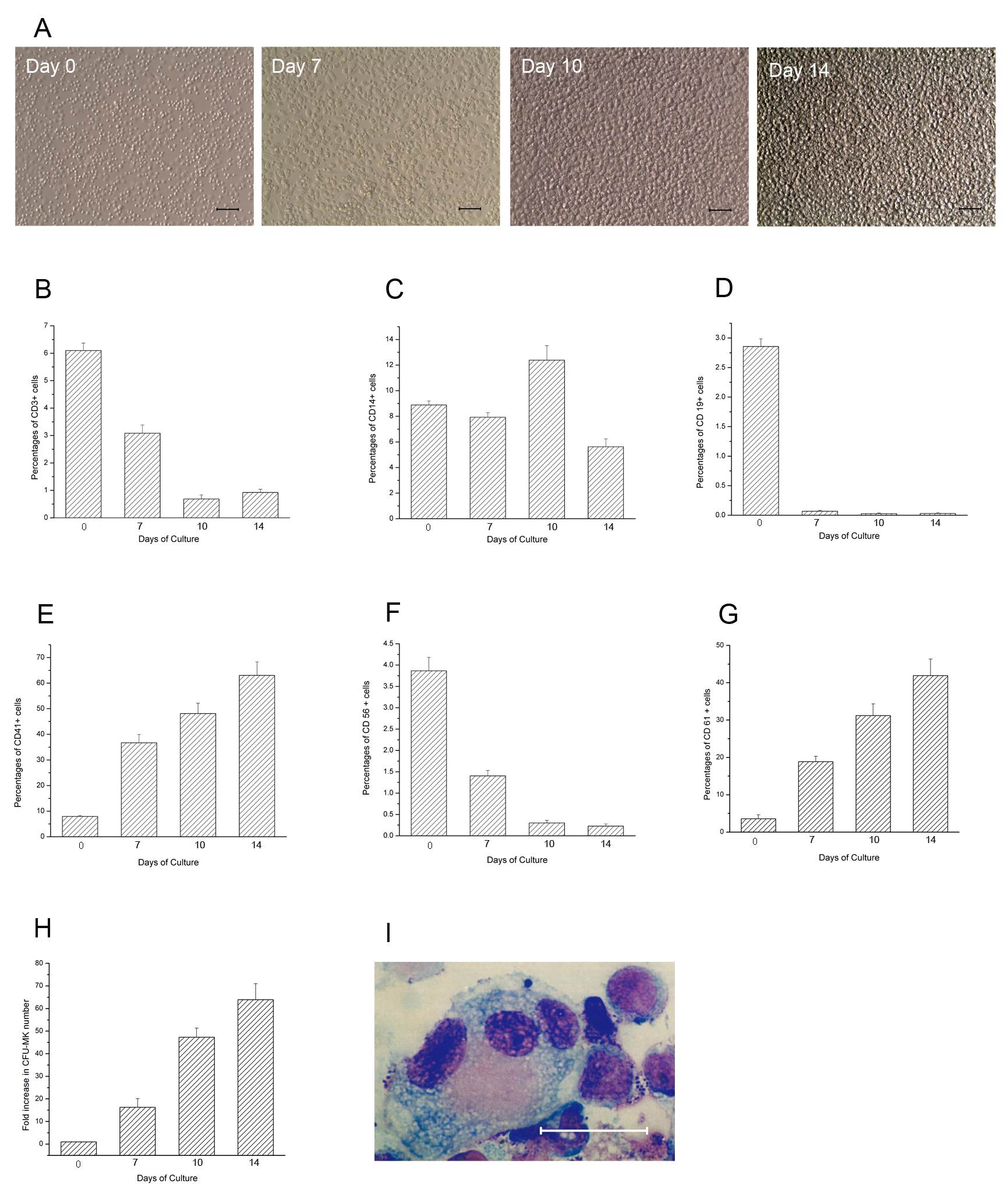

Supplement: Figure S1 — Characterization of megakaryocytic progenitors from cord blood mononuclear cells. (A) Cell morphology of different culture time by microscope observation. Scale bars: 100 µm. Surface marker expression of CD3 (B), CD14 (C), CD19 (D), CD41 (E), CD56 (F), and CD61 (G) in expanded CB population during 14 days of culture. (H) CFU-Mk expansions after 14 days of culture. Data are shown as mean±SD from three experiments. (I) Typical morphology of mature megakaryocytes on day 14 culture was shown. Scale bars: 50 µm. (TIF) [file pone.0054941.s001.tif]

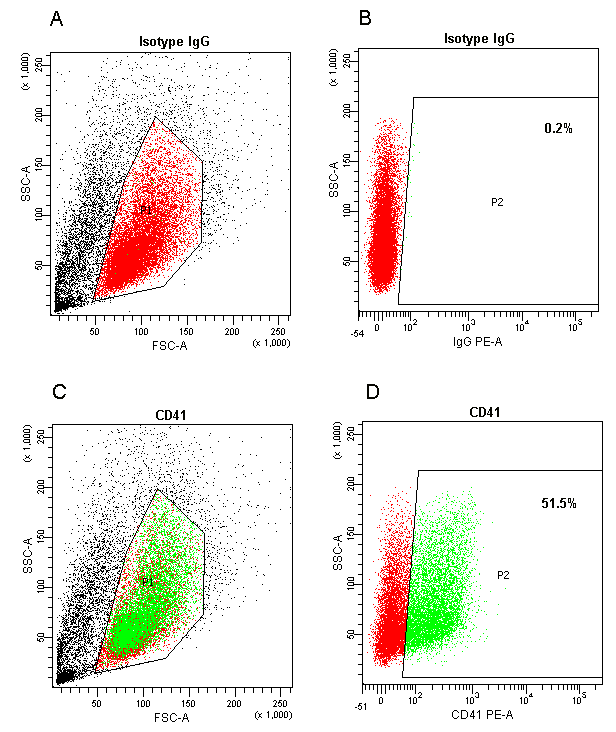

Supplement: Figure S2 — FACS plots showed the cell size and co-staining of CD41+ cells at day 14 culture. (A) and (B) showed the results of isotype IgG. (C) and (D) showed the results of CD41 expression. (TIF) [file pone.0054941.s002.tif]

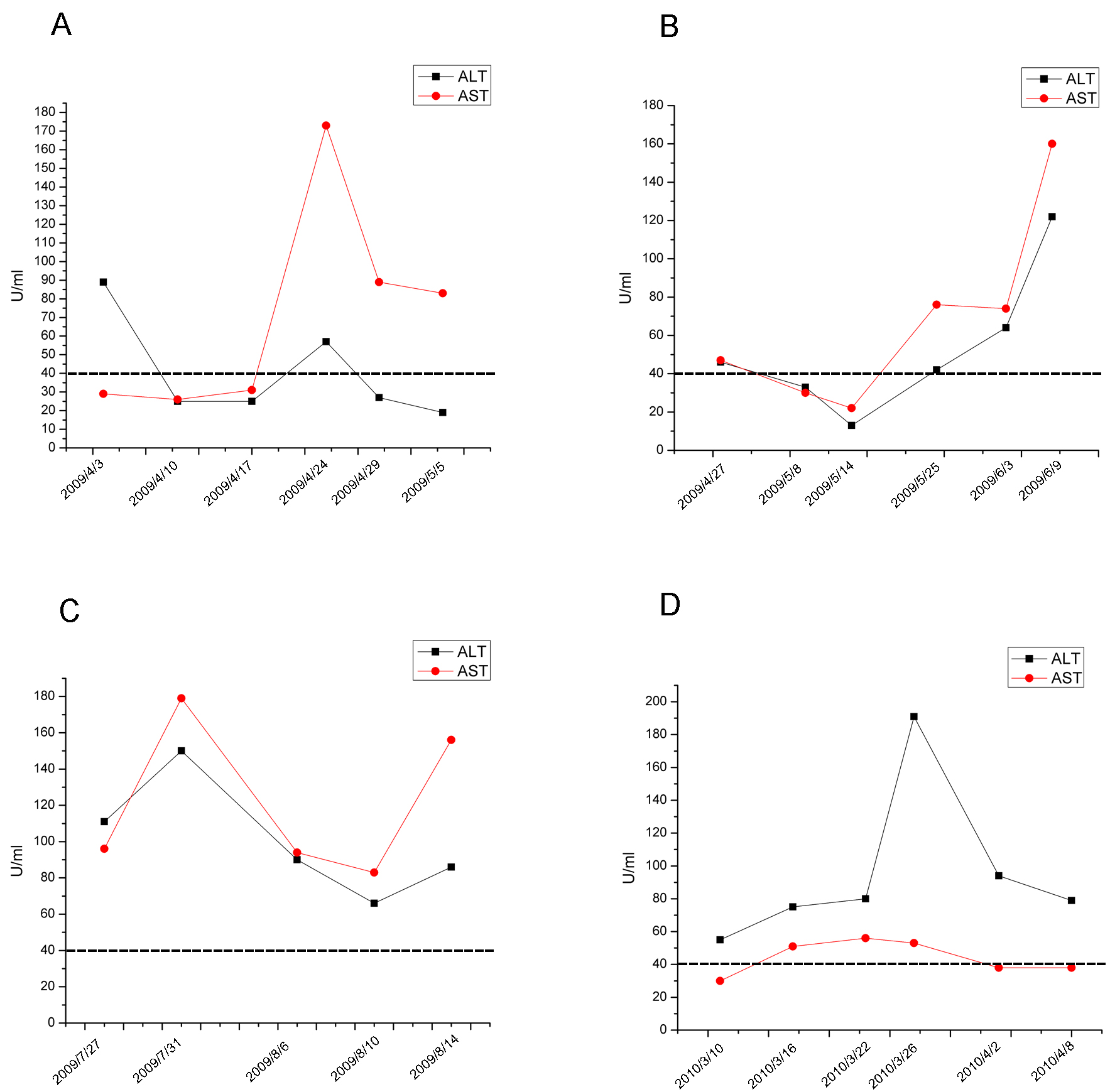

Supplement: Figure S3 — Transaminase changes of four patients before and after MPs infusion. (A) Data of patient 1. The chemotherapy time is 2009/4/7, and the MPs infusion time is 2009/4/14. (B) Data of patient 2. The chemotherapy time is 2009/5/5, and the MPs infusion time is 2009/5/19. (C) Data of patient 7. The chemotherapy time is 2009/7/30, and the MPs infusion time is 2009/8/7. (D) Data of patient 20. The chemotherapy time is 2010/3/12, and the MPs infusion time is 2010/3/23. All the four patients had different degrees of elevated transaminases before MP treatment. Dashed lines shows the normal value of ALT and AST. ALT: alanine transarninase, AST: aspartate aminotransferase. (TIF) [file pone.0054941.s003.tif]
