# Supplementary material for: Infusion of Megakaryocytic Progenitor Products Generated from Cord Blood Hematopoietic Stem/Progenitor Cells: Results of the Phase 1 Study
Source: PLoS One. 2013 Feb 4;8(2):e54941. doi: 10.1371/journal.pone.0054941 (PMC3563646; doi:10.1371/journal.pone.0054941)
Supplement: Table S1 — ABO blood group and HLA typing data of patients and paired cord blood. (DOC) [file pone.0054941.s004.doc]

**Table S1.** HLA typing and ABO blood group of patients and donor cord blood

| Date | No | HLA-A | HLA-B | HLADRB1 | ABO blood group |
| --- | --- | --- | --- | --- | --- |
| 090414 | Patient 1 | 0201/07..  24 | 1301/..  46 | 0901  0901 | A |
|  | CB 1 | 0201/04.07..  0206/10.. | 1518/72..  46 | 0405  1501 | O |
| 090519 | Patient 2 | NA | | | O |
|  | CB 2 |  |  |  | A |
| 090626 | Patient 3 | 24  24 | 4004/06..  5101/03.. | 1201  1403 | A |
|  | CB 3 | 0201/07..  6802/18.. | 1402/09..  4001/22.. | 0102  1101 | B |
| 090710 | Patient 4 | 30  32 | 13  5108 | 0701  1501 | B |
|  | CB4 | 11  24 | 1301/02..  54 | 0405  1405 | B |
| 090721 | Patient 5 | 03  24 | 35  52 | 0901  1501 | AB |
|  | CB5 | 0201/04..  0206/07.10.. | 1301/07..  48 | 1407  1501 | B |
| 090730 | Patient 6 | 0201/07  24 | 4001/11..  4002/29.. | 0901  1501 | B |
|  | CB6 | NA | | | O |
| 090807 | Patient 7 | 01  24 | 37  54 | 0301  0405 | A |
|  | CB7 | 11  30 | 1302/08..  4001/22.. | 0701  1302 | O |
| 090821 | Patient 8 | 0201/07..  33 | 1402/09  5101/03.. | 0102  1403 | B |
|  | CB8 | NA | | | O |
| 090922 | Patient 9 | 11  11 | 4001/10..  46 | 0405  0901 | B |
|  | CB9 | 03  33 | 1402/..  35 | 01  14 | O |
| 091016 | Patient 10 | 01  24 | 4002/29..  4006/70.. | 0803  0901 | O |
|  | CB10 | 0201/04.07..  0201/04/07.. | 46  5507（54） | 0803  1501 | B |
| 091028 | Patient 11 | 24  31 | 4001/22..  5101/03.. | 0401  1101 | B |
|  | CB11 | NA | | | AB |
| 091112 | Patient 12 | 0201/07..  30 | 1302/..  3901/04.. | 0701  0901 | AB |
|  | CB12 | 0201/07..  33 | 57  67 | 0701  1302 | NA |
| 091120 | Patient 13 | 26  31 | 1501/26..  1501/26.. | 0901  1101 | B |
|  | CB13 | 0201/07..  30 | 1302/11..  1501/04.33.. | 0701  0901 | O |
| 091207 | Patient 14 | 0201/04.07..  24 | 4001/22..  54 | 0901  1501 | A |
|  | CB14 | 0201/04/07..  0206/10.. | 1501/05.20..  3501/07.. | 0101  0901 | A |
| 091215 | Patient 15 | 01  11 | 08  1518/.. | 0701  0901 | B |
|  | CB15 | NA | 1501/26..  3802/15.. | 0401  12021 | O |
| 091225 | Patient 16 | 01  30 | 1518/..  4001/22.. | 0403  0901 | B |
|  | CB16 | NA | | | O |
| 100115 | Patient 17 | 0201/07..  11 | 08  52 | 0301  0404 | O |
|  | CB17 | 24  33 | 3501/07..  58 | 0901  1302 | B |
| 100126 | Patient 18 | 0201/07..  31 | 5101/03..  54 | 1405  1501 | O |
|  | CB18 | 11  31 | 1525  5102 | 0405  0802 | O |
| 100208 | Patient 19 | 11  29 | 0705/06..  1502/88.. | 0803  1202 | NA |
|  | CB19 | 0201/07..  11 | 3503/36..  5101/03.. | 1201  1454 | O |
| 100323 | Patient 20 | 11  26 | 3501/04..  4001/22.. | 1101  1501 | NA |
|  | CB20 | 24  24 | 1527  4001/22.. | 0901  1101 | A |
| 100416 | Patient 21 | 0201/04.07..  0201/04.07.. | 46  48 | 0803  0901 | B |
|  | CB21 |  | 4402/11..  52 | 0101  1106 | O |
| 100421 | Patient 22 | 03  31 | 1509/18..  5101/03.. | 0402  0901 | A |
|  | CB22 | 0201/07..  30 | 1301/02..  52 | 0701  0701 | O |
| 100509 | Patient 23 | 0203  03 | 37  67 | 1001  1602 | B |
|  | CB23 | 01  30 | 1302/..  57 | 0701  0701 |  |
| 100524 | Patient 24 | NA | | | B |
|  | CB24 |  |  |  | O |

NA, not applicable
