# Supplementary material for: Infusion of Megakaryocytic Progenitor Products Generated from Cord Blood Hematopoietic Stem/Progenitor Cells: Results of the Phase 1 Study
Source: PLoS One. 2013 Feb 4;8(2):e54941. doi: 10.1371/journal.pone.0054941 (PMC3563646; doi:10.1371/journal.pone.0054941)
Supplement: Protocol S1 — (DOC) [file pone.0054941.s005.doc]

The Tolerance Test of Single Dose Treatment of Thrombocytopenia with "Injection of Umbilical Cord Blood Megakaryocyte Progenitor Cells" after Radiotherapy and Chemotherapy

**1. Objectives of Study**

To evaluate the safety and tolerability of single dose treatment of thrombocytopenia with "Injection of Megakaryocyte Progenitor Cells from Umbilical Cord Blood" after radiotherapy and chemotherapy.

**2. Patients**

Patients with advanced hematological malignancy from Peking University Cancer Hospital & Institute were enrolled in this study.

**Inclusion criteria:** 1. Aged from 18 to 60 years old, open to men and women; 2. within one month after cancer radiotherapy and chemotherapy; 3. ECOG grades 0 or 1，expected survival time is more than or equal to three months; 4. Subjects are thrombocytopenia patients caused by cancer radiotherapy and chemotherapy; 5. platelet, which is more than 20×10^9^/L and less than 60×10^9^/L; 6. Results of liver and renal function are in normal or basic normal situation; 7. There is no infusion contraindication about injection of umbilical cord blood megakaryocyte progenitor cells; 8. understand and sign the informed consent form.

**Exclusion criteria:** 1. thrombocytopenia without cancer radiotherapy and chemotherapy; 2. Patients with primary disease in important organs(liver, kidney), patients accompanied by the immune system disease, serious infections, mental disorders or organ transplant recipients; 3. pregnant, breast-feeding women; 4. Patients did not sign informed consent form; 5. infusion contraindication about injection of umbilical cord blood megakaryocyte progenitor cells.

**2. Treatment Protocols**

MPs from cord blood were infused when the platelet of patients was 20 ≤PLT≤60 (×10^9^/L) after chemotherapy. The interval time between the cord blood megakaryocyte progenitor cell preparation and patient infusion was within two hours. Before MP infusion, the patients was given 10ml 10% calcium gluconate to prevent anaphylaxis. The overvall infusion time was two hours.

**3.** **End points and definitions**

The primary end point was the safety and tolerability of the infusion of cord blood-derived MPs. Patients were monitored after the infusion of MPs for infusional toxicity and adverse events, which were evaluated according to World Health Organization (WHO) criteria. These indicators include fever, anaphylaxis, hemolysis, shock, embolism, hepatolienomegaly, abnormal electrocardiogram, transaminitis, Abnormal renal function, jaundice, vomiting, diarrhea, erythra, GVHD. Secondary end points included platelet recovery after infusion of MPs. The observation period was from the first day of the chemotherapy to beginning time of the next chemotherapy.

组察起止时间是000000000000000000000000000000000000000000000000000000000000000000000000000000000000000000000000000000000000000000000000

**4.Follow-up**

After infusion with cord blood-derived MPs, we conducted one-year follow-up.
