# Supplementary material for: Infusion of Megakaryocytic Progenitor Products Generated from Cord Blood Hematopoietic Stem/Progenitor Cells: Results of the Phase 1 Study
Source: PLoS One. 2013 Feb 4;8(2):e54941. doi: 10.1371/journal.pone.0054941 (PMC3563646; doi:10.1371/journal.pone.0054941)
Supplement: Protocol S2 — (DOC) [file pone.0054941.s006.doc]

**脐带血巨核系祖细胞注射液单次治疗肿瘤化疗后**

**血小板减少的安全性和耐受性研究方案**

**1.临床试验目的：**

评价脐带血巨核系祖细胞注射液单次治疗肿瘤放化疗后血小板减少的安全性和耐受性。

**2. 患者**

患者来自北京大学肿瘤医院，接受化疗后血小板减少患者。

**纳入标准**

1. 年龄18-60岁，男女不限；
2. 肿瘤化疗后1个月内；
3. ECOG分级0或1级，预计生存期≥3月；
4. 受试者为肿瘤放化疗后所致的血小板减少症的患者；
5. 前一个放化疗周期20×10^9^/L≤血小板≤60×10^9^/L，
6. 肝、肾功能结果正常或基本正常；
7. 无脐带血巨核系祖细胞注射液输注禁忌症；
8. 自愿签署知情同意书。

**排除标准**

1. 非肿瘤放化疗后血小板减少；
2. 重要脏器原发疾病者（肝脏、肾脏）、伴有免疫系统疾病、严重感染、精神障碍或器官移植者；
3. 妊娠、哺乳妇女；
4. 患者未签署知情同意书；
5. 脐带血巨核系祖细胞注射液输注禁忌症。

**2. 治疗方案**

肿瘤患者接受化疗后20×10^9^/L≤血小板≤60×10^9^/L的给予单次静脉输注脐带血巨核系祖细胞注射液。脐带血巨核系祖细胞注射液从实验室到病人输注的间隔时间在2小时内，输注前病人给与10%葡萄糖酸钙10ml预防输液反应，输注持续时间2小时，观察病人的生命体征和不良反应。

**3.主要观察治疗指标：**

安全性和耐受性评价，指标包括：生命体征、心电图、发热；过敏；溶血反应；休克；栓塞；肝脾肿大；黄疸；呕吐；腹泻；皮疹；GVHD。血液指标：血常规、血生化。

组察起止时间是000000000000000000000000000000000000000000000000000000000000000000000000000000000000000000000000000000000000000000000000观察起止时间指本治疗周期接受放化疗第一天至下次放化疗开始。

**4. 随访**

接受治疗患者进行为期一年的随访。
